# The impact of sarcopenia on short-term efficacy, toxic and adverse reactions, and prognosis in patients with non-small cell lung cancer undergoing concurrent chemoradiotherapy

**DOI:** 10.3389/fnut.2026.1776203

**Published:** 2026-07-14

**Authors:** Yin Yuan, Zhao Yu, Yanchen Ma, Kaining Wang, Jianming Ding, Zelai He, Gengming Wang

**Affiliations:** Department of Radiotherapy, The First Affiliated Hospital of Bengbu Medical University, Bengbu, China

**Keywords:** concurrent chemoradiotherapy, intermuscular adipose tissue, non-small cell lung cancer, prognosis, sarcopenia, short-term efficacy

## Abstract

**Background:**

Sarcopenia (SP) is an important indicator for evaluating the nutritional status of cancer patients, but its impact on short-term efficacy, toxic and adverse reactions, and long-term survival in patients with non-small cell lung cancer (NSCLC) undergoing concurrent chemoradiotherapy (CRT) remains incompletely understood.

**Methods:**

Clinical data of 86 newly diagnosed NSCLC patients who were admitted to Bengbu Medical University First Affiliated Hospital from January 2020 to June 2024 were collected. The skeletal muscle index (SMI) was calculated based on computed tomography (CT) images at the mid-vertebral body of L3. Patients were divided into the SP group (46 cases, 53.5%) and the non-sarcopenia group (40 cases, 46.5%). The incidence of adverse events, short-term efficacy (per RECIST 1.1 criteria), 1 and 3-year overall survival (OS), and progression-free survival (PFS) were compared between the two groups. Cox proportional hazards regression model was used to analyze prognostic factors.

**Results:**

Follow-up was conducted until July 31, 2025. The objective response rate (ORR) in the sarcopenia group was significantly lower than that in the non-sarcopenia group (56.5 vs. 80.0%, χ^2^ = 5.371, *P* = 0.020), and the complete response rate (CR) showed a decreasing trend (4.3 vs. 20.0%, *P* = 0.055). There was no significant difference in the incidence of severe adverse events between the two groups (*P* > 0.05), but the incidence of radiation pneumonitis was higher in the sarcopenia group (43.5 vs. 25.0%). The median OS of the entire cohort was 24 months (95% confidence interval [CI] 18 ~30 months), and the median OS in the sarcopenia group (19 months) was significantly shorter than that in the non-sarcopenia group (36 months, *P* < 0.001). Multivariate Cox proportional hazards regression analysis revealed that sarcopenia (hazard ratio [HR] = 1.729, 95% CI 1.024–2.910, *P* = 0.040), Karnofsky Performance Status (KPS) score < 80, serum albumin < 40.0 g/L, and intermuscular adipose tissue (IMAT) ≥12.46 cm^2^ were independent prognostic factors for OS.

**Conclusion:**

SP can reduce the short-term efficacy of CRT and shorten OS in NSCLC patients. It serves as an independent prognostic factor for these patients, providing guidance for formulating clinical treatment strategies.

## Introduction

Lung cancer is one of the malignant tumors with the highest incidence and mortality worldwide (1), with NSCLC accounting for 85% of all cases (2). Due to insidious early symptoms, approximately 30% of patients are diagnosed at an advanced local stage (3), for which CR serves as the standard treatment modality (4). However, current clinical prognostic evaluation mainly relies on the TNM staging system, which fails to accurately reflect individual patient differences (e.g., nutritional status, physical reserve). This leads to a lack of individualized basis for formulating treatment strategies, highlighting an urgent need to explore new prognostic predictors.

SP was first proposed by Rosenberg and defined as a syndrome characterized by progressive reduction in skeletal muscle mass and decrease in muscle strength, which ultimately leads to adverse outcomes (5–7). Quantification of skeletal muscle area using CT at L3 is currently the recognized non-invasive diagnostic method (8). Furthermore, existing studies have confirmed that SP is associated with poor prognosis in various malignant tumors, such as hepatocellular carcinoma (9) and colorectal cancer (10), with potential mechanisms involving tumor microenvironment disruption, decreased treatment tolerance, and activation of systemic inflammatory responses (11–13). The incidence of SP in lung cancer patients is approximately 46.8%−55.8% (14, 15), which is significantly higher than that in other cancer types. However, most existing studies have focused on surgically treated patients, and sufficient evidence regarding the comprehensive impact of SP on short-term efficacy, toxic and adverse reactions, and long-term survival in NSCLC patients undergoing CRT remains insufficient. Furthermore, as a key indicator of abnormal fat distribution, whether the synergistic effect of IMAT with SP affects the prognosis of CRT patients remains unclear, and evaluation of this “muscle-fat crosstalk” dimension may provide a new perspective for prognostic prediction.

This study aims to systematically investigate the impact of SP on toxic and adverse reactions, short-term efficacy, OS, and PFS in NSCLC patients during CRT. It also intends to analyze the prognostic value of IMAT, nutritional indicators, and other factors, providing a basis for formulating individualized clinical treatment strategies and improving patient prognosis.

## Materials and methods

### Patients

**Figure 1:**
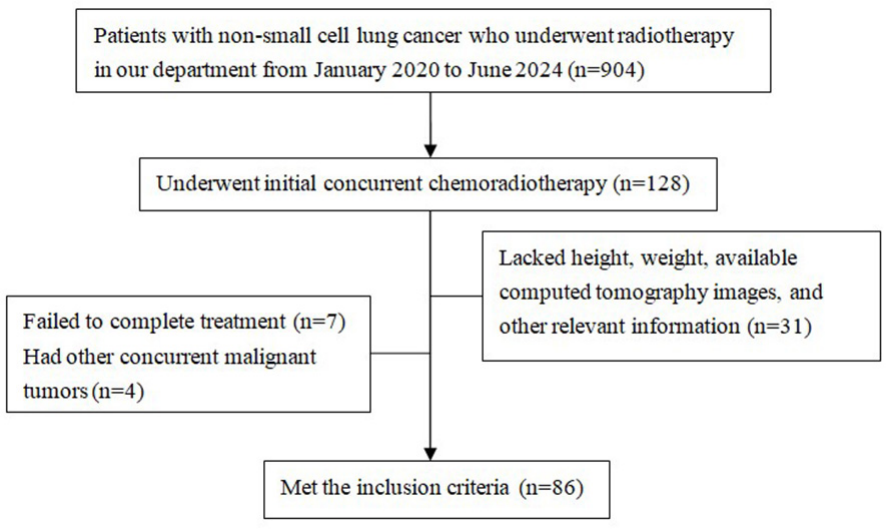
Consort diagram. *N* = number of patients.

Eighty-six pathologically confirmed NSCLC patients who received CRT and were admitted to the First Affiliated Hospital of Bengbu Medical University between January 2020 and June 2024 were included in this study. Inclusion criteria: (1) Male or female, aged ≥18 years with an expected survival time >3 months; (2) Pathologically confirmed NSCLC by histology or cytology; (3) Initial concurrent CRT; (4) KPS score ≥70 (able to tolerate CRT). Exclusion criteria: (1) Previous receipt of other anti-tumor therapies; (2) Presence of other malignant tumors; (3) Distant metastasis; (4) Complications that may affect treatment tolerance or follow-up completeness, such as severe cardio-cerebrovascular diseases, uncontrolled diabetes mellitus, or hyperthyroidism. Clinical staging was determined according to the 8th edition of the American Joint Committee on Cancer (AJCC) NSCLC staging system. The flowchart of patient selection is shown in Figure 1.

### Data collection

Data on the following baseline clinical data were collected: age, gender, smoking history, alcohol consumption history, KPS score, histological subtype, and TNM stage. Baseline nutritional parameters included: body weight, body mass index (BMI), weight loss during CRT, pre-treatment anemia history, serum albumin level, nutritional risk index (NRI), and prognostic nutritional index (PNI). Inflammatory markers included: neutrophil-to-lymphocyte ratio (NLR), platelet-to-lymphocyte ratio (PLR), and lymphocyte-to-monocyte ratio (LMR). Additionally, treatment characteristics were collected, including chemotherapy regimens and radiotherapy doses.

### Treatment

Radiotherapy was delivered using 6 MV X-rays with intensity-modulated and conformal radiotherapy (IMRT) or TomoTherapy (Tomo), with a total dose of 54–64 Gy in 28–32 fractions (single fraction dose: 1.8–2.2 Gy). The target volume encompassed the primary tumor and regional lymph node drainage areas. Chemotherapy was dominated by platinum-based doublet regimens, including paclitaxel + carboplatin, pemetrexed + carboplatin, etc., which were administered at standard doses and cycles.

### Fat/muscle measurement

**Figure 2:**
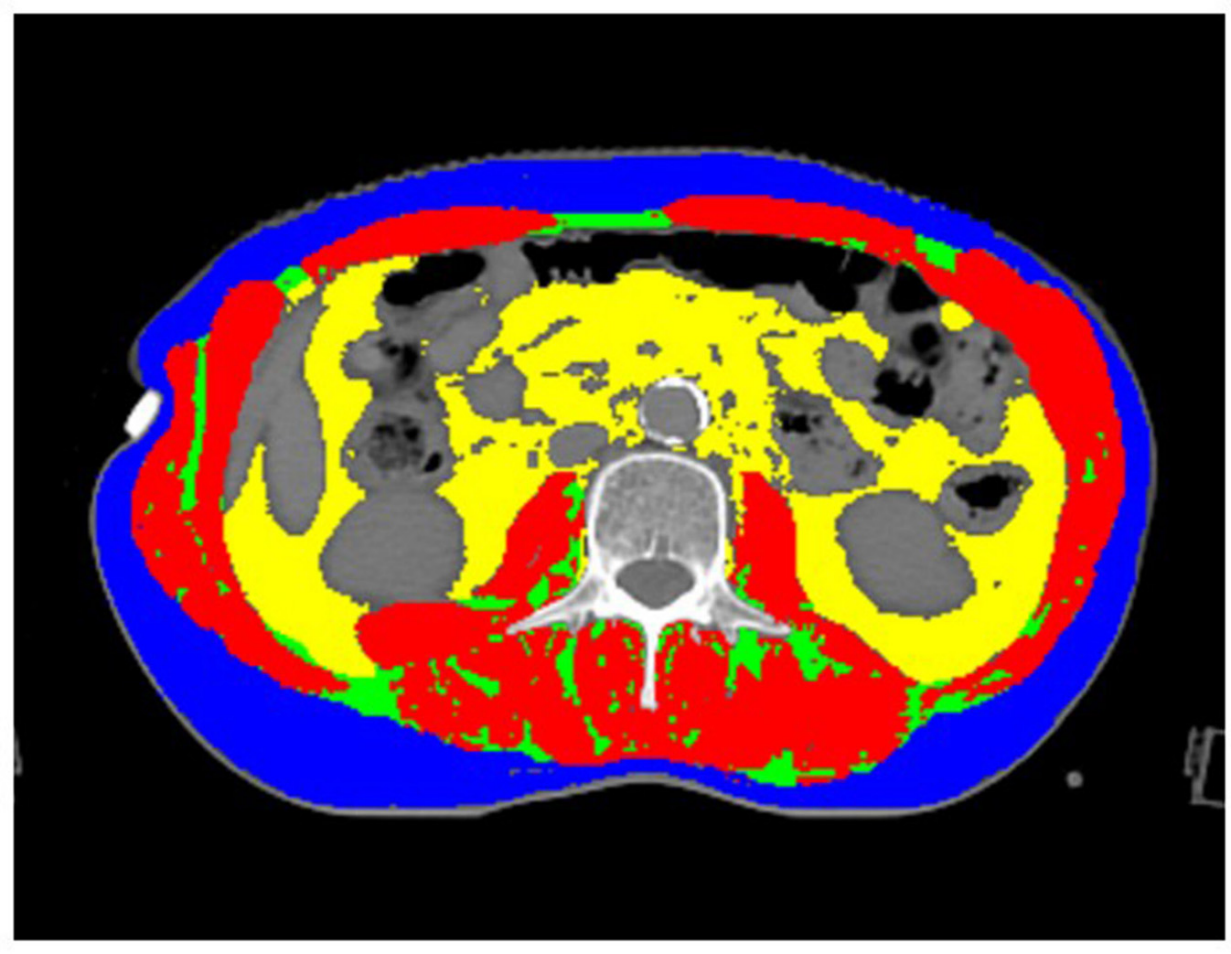
Example of skeletal muscle (red), subcutaneous adipose tissue (blue), visceral adipose tissue (yellow), and intermuscular adipose tissue (green), measured on the axial image of the L3 vertebra.

Slice-o-matic 5.0 software (Tomovision, Montreal, Canada) was used to analyze CT images at the mid-vertebral body of L3. Threshold settings were as follows: skeletal muscle (−29 to 150 HU), subcutaneous adipose tissue (−190 to −30 HU), visceral adipose tissue (−150 to −50 HU), and IMAT (−190 to −30 HU), as illustrated in Figure 2. The SMI was calculated as the cross-sectional area of skeletal muscle (cm^2^) divided by height squared (m^2^). IMAT was not normalized to height squared, and statistical analysis was performed using the quantitatively measured raw cross-sectional area at the mid-L3 vertebral body CT level, with the unit of cm^2^. The diagnostic criteria for sarcopenia were based on the thresholds proposed by Martin et al. (16): SMI ≤ 41 cm^2^/m^2^ for females regardless of BMI; SMI ≤ 53 cm^2^/m^2^ for males with BMI ≥25 kg/m^2^, and SMI ≤ 43 cm^2^/m^2^ for males with BMI < 25 kg/m^2^. This standard is the most widely used tumor-specific criterion for CT-based sarcopenia assessment in solid tumors, including NSCLC. In contrast, the criteria of the Asian Working Group for Sarcopenia (AWGS) were primarily developed for community-dwelling healthy older adults and do not account for the characteristics of cancer cachexia-associated secondary sarcopenia, making them unsuitable for the study population.

### Clinical definitions

According to Chinese criteria (17), anemia was defined as a serum hemoglobin level ≤ 12 g/dl in females and ≤ 13 g/dl in males. Patients were divided into the normal group and the hypoalbuminemia group based on the cutoff value of serum albumin (40.0 g/L) in our laboratory.

The PNI was calculated as follows: serum albumin (g/L) + [5 × peripheral blood lymphocyte count ( × 10^9^/L)]. The NRI was calculated as: [1.519 × albumin (g/dl)] + 41.7 × (current weight/ideal weight). NRI is used to assess the degree of nutritional risk, and PNI is used to predict treatment tolerance and prognosis in cancer patients. Both are commonly used clinical nutritional assessment tools (18, 19).

### Evaluation of adverse events and treatment response

Adverse events during treatment were recorded and evaluated using the Common Terminology Criteria for Adverse Events (CTCAE) version 5.0. One month after the completion of CRT, treatment response assessment was performed in accordance with the Response Evaluation Criteria in Solid Tumors (RECIST) version 1.1.

### Follow-up

Patients underwent the first reexamination 1 month after CRT, followed up every 3 months for 2 years, and then every 6 months thereafter, with follow-up concluding on July 31, 2025. The primary endpoint was OS, defined as the time from the initiation of CRT to patient death or the last follow-up. The secondary endpoint was PFS, defined as the time from the initiation of CRT to disease recurrence, metastasis, or the last follow-up date.

### Statistical analysis

Statistical analyses were performed using SPSS 27.0.1 (IBM Corporation, Armonk, NY, USA). Comparisons of categorical data were conducted using the Pearson χ^2^ test, corrected χ^2^ test, or Fisher's exact test based on theoretical frequencies. For continuous data, the independent samples *t*-test was applied if normally distributed; otherwise, the Mann-Whitney *U*-test was used. Survival curves were plotted using the Kaplan-Meier method, and intergroup differences were compared with the Log-rank test. Univariate Cox regression analysis was performed to screen for potential prognostic factors (*P* < 0.1), which were subsequently included in the multivariate Cox regression model (adopting the stepwise regression method). Multicollinearity was excluded via the variance inflation factor test. All tests were two-tailed, and a *P*-value < 0.05 was considered statistically significant. The cutoff value of IMAT at 12.46 cm^2^ was determined by median dichotomization of IMAT values in the study cohort. This is the most widely adopted grouping method for continuous variables in oncological body composition research, which effectively balances sample sizes between groups, avoids data-driven overfitting caused by outcome-dependent optimal cutoffs, and yields more reproducible results.

## Results

### Patient characteristics

A total of 86 patients were included in the study, among whom 46 (53.5%) were in the sarcopenia group and 40 (46.5%) in the non-sarcopenia group. There were 76 males (88.4%), 68 cases of squamous cell carcinoma (79.1%), with a median age of 67 years (range: 40–84 years). Except for the SMI, there were no significant differences in baseline clinical characteristics (e.g., gender, pathological type, TNM stage; Table 1) or inflammatory markers (NLR, PLR, LMR; Table 2) between the two groups (all *P* > 0.05), indicating comparability. The SMI in the SP group was significantly lower than that in the non-sarcopenia group (41.06 ± 5.36 vs. 49.77 ± 5.60 cm^2^/m^2^, *P* < 0.001).

**Table 1:** Comparison between sarcopenic and non-sarcopenic patients [*n* (%) or mean ± SD or median (Q1, Q3)].

Characteristics	Sarcopenic	Non-sarcopenic	χ^2^	*P*
	*n* = 46 (0.53)	*n* = 40 (0.47)		
Age, years			0.029	0.864
≤ 67	25 (54.3)	21 (52.5)		
>67	21 (45.7)	19 (47.5)		
Gender			0.000	1.000
Male	41 (89.1)	35 (87.5)		
Female	5 (10.9)	5 (12.5)		
KPS score				1.000
< 80	3 (6.5)	2 (5.0)		
≥80	43 (93.5)	38 (95.0)		
Smoking			2.166	0.141
Yes	28 (60.9)	18 (45.0)		
No	18 (39.1)	22 (55.0)		
Alcohol			0.002	0.965
Yes	14 (30.4)	12 (30.0)		
No	32 (69.6)	28 (70.0)		
BMI, kg/m^2^			1.951	0.163
< 18.5	4 (8.7)	0 (0.0)		
≥18.5	42 (91.3)	40 (100.0)		
Albumin, g/L			3.369	0.066
< 40.0	34 (73.9)	22 (55.0)		
≥40.0	12 (26.1)	18 (45.0)		
Anemia			0.748	0.387
Yes	25 (54.3)	18 (45.0)		
No	21 (45.7)	22 (55.0)		
NRI			0.532	0.466
< 100	11 (23.9)	7 (17.5)		
≥100	35 (76.1)	33 (82.5)		
PNI	54.85 (49.94, 60.88)	54.95 (48.57, 64.14)		0.979
Histological subtype			0.039	0.843
Squamous cell carcinoma	36 (78.3)	32 (80.0)		
Adenocarcinoma	10 (21.7)	8 (20.0)		
TNM			1.945	0.378
IIIA	14 (30.4)	7 (17.5)		
IIIB	23 (50.0)	24 (60.0)		
IIIC	9 (19.6)	9 (22.5)		
Weight loss, %			0.001	0.978
< 5	39 (84.8)	34 (85.0)		
≥5	7 (15.2)	6 (15.0)		
Nutrition support			1.268	0.260
Yes	10 (21.7)	5 (12.5)		
No	36 (78.3)	35 (87.5)		
Chemotherapy regimens			0.968	0.325
Platinum-containing	44 (95.7)	35 (87.5)		
Others	2 (4.3)	5 (12.5)		
Dose, Gy			0.235	0.628
< 60	6 (13.0)	3 (7.5)		
≥60	40 (87.0)	37 (92.5)		
SMI, cm^2^/m^2^	41.06 ± 5.36	49.77 ± 5.60		**< 0.001**
SMD, HU	32.03 ± 7.23	32.65 ± 6.23		0.673
SATI, cm^2^/m^2^	27.51 (16.06, 33.21)	22.60 (16.99, 34.13)		0.782
VATI, cm^2^/m^2^	41.03 (22.77, 57.37)	28.96 (12.78, 40.61)		0.059
IMAT, cm^2^	12.46 (7.75, 18.22)	12.33 (8.51, 18.14)		0.788

Bold values indicates the *P*-value of less than 0.05.

**Table 2:** Comparison of inflammatory indicators [Median (Q1, Q3)].

Inflammatory index	Sarcopenic	Non-sarcopenic	Z	*P*
	*n* = 46 (0.53)	*n* = 40 (0.47)		
Neutrophil	4.16 (3.31, 6.07)	4.26 (3.56, 5.41)	−0.061	0.952
Lymphocyte	1.59 (1.25, 1.99)	1.56 (1.37, 1.85)	−0.307	0.759
Platelet	274.50 (184.00, 356.00)	246.00 (180.00, 308.50)	−1.307	0.191
NLR	2.81 (1.92, 4.28)	2.57 (2.00, 3.69)	−0.658	0.511
PLR	167.06 (136.54, 221.74)	139.71 (94.30, 213.81)	−1.195	0.232
LMR	2.88 (1.83, 3.68)	3.13 (2.28, 4.56)	−1.359	0.174

### Evaluation of adverse events and treatment response

Treatment-related adverse events during CRT are shown in Table 3. There was no statistically significant difference in the incidence of severe adverse events between the two groups. Radiation pneumonitis (43.5 vs. 25.0%) was more common in patients with SP than in those without. The ORR in the SP group was significantly lower than that in the non-sarcopenia group (56.5 vs. 80.0%, *P* = 0.020). In addition, the CR rate showed a decreasing trend in the SP group (4.3 vs. 20.0%, *P* = 0.055), as shown in Table 4.

**Table 3:** Comparison of adverse events [*n* (%)].

Adverse events	Sarcopenic *n* = 46 (0.53)			Non-sarcopenic *n* = 40 (0.47)			χ^2^	*P*
	≥3 grade	<3 grade	None	≥3 grade	<3 grade	None		
Leukopenia	19 (41.3)	20 (43.5)	7 (15.2)	12 (30.0)	22 (55.0)	6 (15.0)	1.186	0.276
Neutrophilic granulocytopenia	18 (39.1)	16 (34.8)	12 (26.1)	12 (30.0)	18 (45.0)	10 (25.0)	0.785	0.376
Thrombocytopenia	4 (8.7)	7 (15.2)	35 (76.1)	0 (0.0)	9 (22.5)	31 (77.5)	1.951	0.163
Fatigue	0 (0.0)	6 (13.0)	40 (87.0)	0 (0.0)	2 (5.0)	38 (95.0)		
Nausea/vomiting	4 (8.7)	12 (26.1)	30 (65.2)	1 (2.5)	8 (20.0)	31 (77.5)	0.582	0.446
Radiation pneumonitis	4 (8.7)	16 (34.8)	26 (56.5)	1 (2.5)	9 (22.5)	30 (75.0)	0.582	0.446
Radiation esophagitis	8 (17.4)	10 (21.7)	28 (60.9)	2 (5.0)	14 (35.0)	24 (60.0)	2.105	0.147
Radiation dermatitis	0 (0.0)	11 (23.9)	35 (76.1)	0 (0.0)	5 (12.5)	35 (87.5)		
Pulmonary infection	2 (4.4)	15 (32.6)	29 (63.0)	2 (5.0)	8 (20.0)	30 (75.0)	0.000	1.000

**Table 4:** Comparison of response evaluation [*n* (%)].

Groups	CR	PR	SD	PD	ORR
Sarcopenic (*n* = 46)	2 (4.3)	24 (52.2)	17 (37.0)	3 (6.5)	26 (56.5)
Non-sarcopenic (*n* = 40)	8 (20.0)	24 (60.0)	7 (17.5)	1 (2.5)	32 (80.0)
χ^2^	3.691				5.371
*P*	0.055				0.020

### Survival analysis

**Figure 3:**
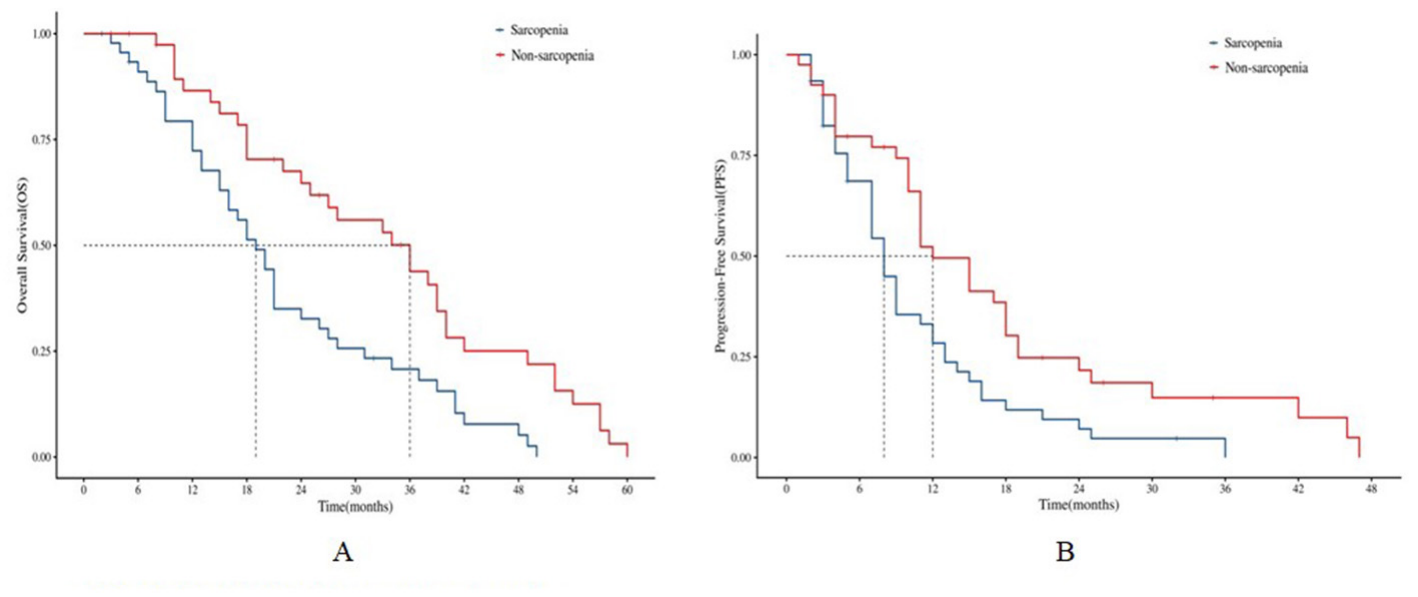
Kaplan–Meier survival curves. **(A)** OS of patients, log-rank χ^2^ = 12.364, *P* < 0.001; **(B)** PFS of patients, log-rank χ^2^ = 7.826, *P* = 0.005.

The median OS of the entire cohort was 24 months. As shown in Figure 3A, the median OS in the sarcopenia (SP) group was 19 months (1-year OS rate: 72.3%, 3-year OS rate: 19.0%), which was significantly shorter than that in the non-sarcopenia group (36 months; 1-year OS rate: 85.6%, 3-year OS rate: 43.8%), with a statistically significant difference (Log-rank χ^2^ = 12.364, *P* < 0.001). The median PFS of the entire cohort was 10 months. As shown in Figure 3B, the median PFS in the SP group was 8 months (1-year PFS rate: 28.4%, 3-year PFS rate: 0.00%), which was significantly shorter than that in the non-sarcopenia group (12 months; 1-year PFS rate: 49.5%, 3-year PFS rate: 12.4%), with a statistically significant difference (Log-rank χ^2^ = 7.826, *P* = 0.005).

### Univariate and multivariate analyses of OS

Univariate analysis showed (Table 5) that KPS score < 80 (6.410, 95% CI 2.445–16.667, *P* < 0.001), serum albumin < 40.0 g/L (HR = 2.659, 95% CI 1.618–4.386, *P* < 0.001), NRI < 100 (HR = 1.773, 95% CI 1.031–3.047, *P* = 0.038), IMAT ≥12.46 cm^2^ (HR = 1.908, 95% CI 1.175–3.096, *P* = 0.009), and the presence of SP (HR = 2.227, 95% CI 1.361–3.636, *P* = 0.001) were adverse prognostic factors for OS. Multivariate analysis demonstrated (Table 6) that KPS score < 80 (HR = 3.027, 95% CI 1.083–8.461, *P* = 0.035), serum albumin < 40.0 g/L (HR = 2.127, 95% CI 1.240–3.647, *P* = 0.006), IMAT ≥12.46 cm^2^ (HR = 1.802, 95% CI 1.080–3.003, *P* = 0.024), and SP (HR = 1.729, 95% CI 1.024–2.910, *P* = 0.040) were associated with shorter OS.

**Table 5:** Univariate analysis of OS and PFS was performed.

Characteristics	OS			PFS		
	HR	95% CI	*P*	HR	95% CI	*P*
Age (>64/ ≤ 64)	1.048	0.662–1.659	0.842	0.776	0.489–1.230	0.280
Sex (Male/Female)	0.779	0.387–1.568	0.484	1.006	5.000–2.026	0.986
KPS (≥80/ < 80)	6.410	2.445–16.667	**< 0.001**	3.831	1.458–10.101	**0.006**
Smoking (No/Yes)	1.293	0.816–2.407	0.273	1.073	0.675–1.705	0.766
Alcohol (No/Yes)	1.358	0.822–2.242	0.232	1.089	0.655–1.809	0.743
BMI (≥18.5/ < 18.5)	1.317	0.408–4.247	0.645	0.546	0.172–1.740	0.307
Weight loss (< 5%/≥5%)	0.781	0.425–1.434	0.425	0.902	0.493–1.651	0.738
Anemia (No/Yes)	1.385	0.877–2.187	0.162	1.269	0.802–2.009	0.309
Albumin (≥40.0/ < 40.0)	2.659	1.618–4.386	**< 0.001**	3.096	1.770–5.405	**< 0.001**
NRI (≥100/ < 100)	1.773	1.031–3.047	**0.038**	1.138	0.661–1.961	0.640
LMR (< 3.03/≥3.03)	0.898	0.569–1.416	0.643	1.167	0.704–1.840	0.506
NLR (< 2.65/≥2.65)	1.326	0.840–2.095	0.226	0.840	0.532–1.326	0.454
PLR (< 162/≥162)	1.261	0.799–1.989	0.319	1.217	0.771–1.919	0.399
PNI (< 54.95/≥54.95)	0.840	0.532–1.325	0.452	1.132	0.717–1.785	0.595
Comorbidities (No/Yes)	1.081	0.663–1.765	0.754	1.521	0.924–2.504	0.099
Nutritional grade (normal/mild malnutrition)	1.145	0.708–1.853	0.582	1.299	0.802–2.105	0.287
Primary tumor location (peripheral/central)	1.507	0.901–2.522	0.118	1.307	0.791–2.160	0.296
Histological subtype (adenocarcinoma/squamous cell carcinoma)	0.845	0.484–1.476	0.553	1.064	0.606–1.867	0.829
*N* (0–1/2–3)	1.101	0.621–1.953	0.742	0.690	0.387–1.230	0.208
Radiotherapy technique (IMRT/TOMO)	1.451	0.773–2.724	0.246	0.375	0.195–0.720	**0.003**
Dose (< 60/≥60)	0.823	0.408–1.657	0.585	0.828	0.410–1.670	0.597
Chemotherapy regimens (platinum-containing/Others)	0.983	0.425–2.273	0.968	0.875	0.379–2.023	0.755
Nutritional support (No/Yes)	0.816	0.438–1.521	0.522	0.947	0.508–1.764	0.863
Consolidation chemotherapy (No/Yes)	1.190	0.663–2.139	0.560	2.132	1.152–3.948	**0.016**
SP (No/Yes)	2.227	1.361–3.636	**0.001**	1.886	1.177–3.022	**0.008**
SMD (< 32.15/≥32.15)	1.548	0.969–2.472	0.068	1.325	0.839–2.091	0.227
IMAT (< 12.46/≥12.46)	1.908	1.175–3.096	**0.009**	0.846	0.536–1.334	0.471
SATI (< 25.46/≥25.46)	0.700	0.443–1.107	0.127	1.131	0.714–1.790	0.599
VATI (< 32.22/≥32.22)	0.757	0.480–1.193	0.230	0.839	0.529–1.329	0.454

Bold values indicates the *P*-value of less than 0.05. Reference group: no sarcopenia; KPS score ≥80; albumin ≥40.0 g/L; IMAT < 12.46 cm^2^; IMRT; no consolidation chemotherapy. HR >1 indicates an adverse prognostic factor.

**Table 6:** Multivariate analysis of OS and PFS.

Characteristics	OS			PFS		
	HR	95% CI	*P*	HR	95% CI	*P*
KPS (≥80/ < 80)	3.027	1.083–8.461	**0.035**	2.880	1.065–7.794	**0.037**
Albumin (≥40.0/ < 40.0)	2.127	1.240–3.647	**0.006**	2.111	1.123–3.966	**0.020**
NRI (≥100/ < 100)	1.308	0.720–2.376	0.378			
SP (No/Yes)	1.729	1.024–2.918	**0.040**	1.738	1.064–2.837	**0.027**
IMAT (< 12.46/≥12.46)	1.802	1.080–3.003	**0.024**			
Consolidation chemotherapy (No/Yes)				1.634	0.810–3.294	0.170
Radiotherapy technique (IMRT/TOMO)				2.002	1.035–3.873	**0.039**

Bold values indicates the *P*-value of less than 0.05. Reference group: no sarcopenia; KPS score ≥80; albumin ≥40.0 g/L; IMAT < 12.46 cm^2^; IMRT; no consolidation chemotherapy. HR >1 indicates an adverse prognostic factor.

### Univariate and multivariate analyses of PFS

Univariate analysis showed (Table 5) that KPS score < 80 (HR = 3.831, 95% CI 1.458–10.101, *P* = 0.006), serum albumin < 40.0 g/L (HR = 3.096, 95% CI 1.770–5.405, *P* < 0.001), receipt of consolidation chemotherapy (HR = 2.132, 95% CI 1.152–3.948, *P* = 0.016), receipt of Tomo (HR = 0.375, 95% CI 0.195–0.720, *P* = 0.003), and the presence of SP (HR = 1.886, 95% CI 1.177–3.022, *P* = 0.008) were adverse prognostic factors for PFS. Multivariate analysis demonstrated (Table 6) that KPS score < 80 (HR = 2.880, 95% CI 1.065–7.794, *P* = 0.037), serum albumin < 40.0 g/L (HR = 2.111, 95% CI 1.123–3.966, *P* = 0.020), receipt of Tomo (HR = 2.002, 95% CI 1.035–3.873, *P* = 0.039), and SP (HR = 1.738, 95% CI 1.064–2.837, *P* = 0.027) were associated with shorter PFS.

## Discussion

This study found that the incidence of radiation pneumonitis in the sarcopenia group (43.5%) was higher than that in the non-sarcopenia group (25.0%). Though it did not reach statistical significance, it suggests that SP may increase the risk of lung injury. This phenomenon may be related to decreased pulmonary function reserve in patients with SP and increased secretion of inflammatory factors (e.g., IL-6, TNF-α) caused by skeletal muscle reduction (11), and the local inflammatory microenvironment exacerbates radiotherapy-induced lung tissue damage. Meanwhile, the ORR in the SP group was significantly lower than that in the non-sarcopenia group (56.5 vs. 80.0%, *P* = 0.020), and the CR rate showed a decreasing trend (4.3 vs. 20.0%, *P* = 0.055), indicating that SP may ultimately affect the anti-tumor efficacy of CRT by impairing the body's anti-tumor immunity and reducing treatment tolerance. Multivariate analysis further confirmed that SP, KPS score < 80, serum albumin < 40.0 g/L, and IMAT≥12.46 cm^2^ were independent prognostic factors for OS, highlighting the importance of the “nutrition-muscle-fat” three-dimensional assessment in prognostic prediction for CRT patients.

The clinical significance of SP in the field of radiation therapy (RT) has been increasingly recognized. On one hand, some side effects of RT (such as nausea, diarrhea, esophagitis, taste changes, and xerostomia) are believed to negatively affect appetite, thereby leading to SP through reduced nutrition. On the other hand, SP exerts a significant adverse effect on the OS of patients receiving radiotherapy, which has been documented in thoracic and gastrointestinal cancers including non-small cell lung cancer, esophageal cancer, and rectal cancer (20–24). A study on the prognostic impact of SP in NSCLC patients undergoing CRT found that after CRT, both BMI and SMI of patients significantly decreased (*P* < 0.001), and the survival time of SP patients was significantly shorter than that of non-SP patients (25). This indicates that appropriate support to prevent skeletal muscle loss during CRT may help improve the prognosis of esophageal cancer patients. An analysis of 328 NSCLC patients who underwent surgery by Nakamura et al. (15) also confirmed that SP is an independent risk factor for decreased 5-year OS and the only pre-operative factor that can be improved through intervention. Unlike the aforementioned studies, the present study focuses on the CRT population, filling the research gap regarding SP in the non-surgical standard treatment of NSCLC. It clearly confirms that SP is an independent prognostic indicator for OS and PFS in CRT patients, providing a new biomarker for prognostic stratification of this population.

Regarding the association between sarcopenia and treatment-related toxicity during CRT in cancer patients, existing studies have yielded inconsistent conclusions, mainly due to differences in tumor types, treatment regimens, and study designs. Martin et al. (16) demonstrated that sarcopenia is associated with increased chemotherapy toxicity and decreased treatment tolerance in patients with various solid tumors including lung cancer; while Olmez et al. (26) only reported the negative impact of sarcopenia on pathologic complete response, and did not involve treatment toxicity-related analysis in rectal cancer patients receiving neoadjuvant concurrent chemoradiotherapy. In the present study, there was no significant difference in the incidence of serious adverse events between the two groups, but the efficacy in the SP group was significantly reduced, suggesting that the impact of SP on NSCLC patients undergoing CRT may focus more on “efficacy impairment” rather than “toxicity enhancement”. This discrepancy may be related to tumor type, treatment regimens (e.g., chemotherapy drug selection, radiotherapy dose), and patient baseline status: NSCLC CRT is mainly based on platinum-based doublet regimens combined with thoracic radiotherapy. Insufficient muscle mass in SP patients may mainly affect drug metabolism efficiency and anti-tumor immune response, rather than directly exacerbating acute toxicity such as mucosal injury. This speculation requires further verification by future multicenter studies.

The association between inflammation and SP has been widely reported, but no significant differences in inflammatory markers (NLR, PLR, LMR) were observed between the two groups in this study (all *P* > 0.05). This result may be related to the following factors: NLR, PLR, and other similar markers are systemic inflammatory indicators, while SP-related inflammation is mainly characterized by local microenvironmental disorders (e.g., accumulation of pro-inflammatory factors in muscle tissue), making systemic markers insufficient to fully reflect this condition; this study did not dynamically monitor changes in inflammatory indicators during treatment, which may have missed the temporal association between SP and inflammation. Future studies may combine local inflammatory factors such as IL-6 and TNF-α to more comprehensively explore the inflammation-related mechanisms of SP (27, 28).

As a classic nutritional status indicator, the association between serum albumin and the prognosis of cancer patients has been fully confirmed (29). Both univariate and multivariate analyses in this study demonstrated that serum albumin < 40.0 g/L is an independent adverse prognostic factor for OS and PFS, which is consistent with the findings of Qian et al. (30) in esophageal cancer patients undergoing CRT. This result further emphasizes that nutritional support (e.g., albumin supplementation, individualized nutritional intervention) may serve as an important adjuvant measure to improve the prognosis of CRT patients, especially for the high-risk population with SP combined with hypoalbuminemia.

Notably, this study found that IMAT≥12.46 cm^2^ is an independent prognostic factor for OS in NSCLC patients after CRT (HR = 1.802, 95% CI 1.080–3.003, *P* = 0.024), suggesting that abnormal fat distribution may synergistically affect patient prognosis with SP. This result naturally links to the clinical phenotype of “sarcopenic obesity (SO)”—SO is characterized by “skeletal muscle reduction + excessive fat accumulation”, and elevated IMAT is one of the key manifestations of abnormal fat distribution in SO patients (31). Unlike this study focusing on IMAT, a retrospective analysis of gastric cancer patients receiving adjuvant chemoradiotherapy by Li et al. (32) showed that SO, rather than SP, is an independent prognostic predictor, indicating that the synergistic effect of abnormal fat and muscle reduction may be more important than a single factor in some tumor types. Stangl-Kremser et al. (33) also confirmed in urothelial carcinoma patients that SO is significantly associated with cancer-specific survival rate (HR = 5.095, 95% CI 1.4–16.7, *P* = 0.01), further highlighting the value of the interaction between muscle and fat in tumor prognosis assessment. Combined with the results of this study, it is speculated that as a key fat indicator related to SO, elevated IMAT may exacerbate the local inflammatory microenvironment and reduce treatment tolerance, thereby jointly worsening the prognosis of NSCLC patients with SP. This also provides a basis for the subsequent exploration of a “combined muscle-fat prognostic assessment model”.

Previous studies have shown that Tomo is superior to intensity-modulated radiotherapy (IMRT) in terms of target volume accuracy and normal tissue sparing (34). However, multivariate analysis in this study revealed that patients who received Tomo therapy had shorter PFS (HR = 2.002, 95% CI 1.035–3.873, *P* = 0.039). This contradictory result may be related to selection bias: in this study, patients who received Tomo therapy were mostly high-risk individuals with larger tumor volume, complex target volume (e.g., invasion of mediastinal large blood vessels), or complicated with poor pulmonary function, who had a higher inherent prognostic risk. Additionally, the small number of Tomo-treated cases resulted in limited statistical power. Future studies need to expand the sample size and conduct prospective research to further verify the efficacy and safety of Tomo in high-risk NSCLC patients.

This study has the following limitations: This study is a single-center retrospective study with a small sample size (86 cases), which may introduce selection bias. To verify the robustness of the SP diagnostic criterion selection, we reclassified all patients using the AWGS 2014 criteria. The results showed no significant difference in patient distribution between groups, and the prognostic impact of sarcopenia on OS and PFS remained consistent with the original analysis, indicating that the choice of diagnostic criteria did not alter the core conclusions of this study; The changes in SMI and IMAT before and after treatment were not dynamically monitored, so the impact of SP improvement on prognosis cannot be assessed; Confounding factors that may affect the occurrence and prognosis of SP, such as nutritional intervention and exercise status, were not included, and their regulatory roles remain unclear; A small number of patients received Tomo therapy, so the results need to be interpreted with caution. Future multicenter prospective studies can be conducted to explore the effect of SP intervention strategies (e.g., nutritional support + resistance training) on improving the prognosis of NSCLC patients undergoing CRT, and to verify the clinical utility of the “combined muscle-fat prognostic assessment model”, so as to provide more solid evidence for the realization of individualized treatment.

## Data Availability

The original contributions presented in the study are included in the article/supplementary material, further inquiries can be directed to the corresponding author.

## References

[1] Siegel RL Giaquinto AN Jemal A. Cancer statistics 2024. CA Cancer J Clin. (2024) 74:12–49. doi: 10.3322/caac.21820 38230766

[2] Detterbeck FC Boffa DJ Kim AW Tanoue LT. The eighth edition lung cancer stage classification. Chest. (2017) 151:193–203. doi: 10.1016/j.chest.2016.10.010 27780786

[3] Kim HC Ji W Lee JC Kim HR Song SY Choi CM. Prognostic factor and clinical outcome in stage III non-small cell lung cancer: a study based on real-world clinical data in the Korean population. Cancer Res Treatment. (2021) 53:1033–41. doi: 10.4143/crt.2020.1350 33592139 PMC8524024

[4] Conibear J. Rationale for concurrent chemoradiotherapy for patients with stage III non-small-cell lung cancer. Br J Cancer. (2020) 123:10–7. doi: 10.1038/s41416-020-01070-6 33293671 PMC7735212

[5] Chen LK Liu LK Woo J Assantachai P Auyeung TW Bahyah KS . Sarcopenia in Asia: consensus report of the Asian working group for sarcopenia. J Am Med Dir Assoc. (2014) 15:95–101. doi: 10.1016/j.jamda.2013.11.025 24461239

[6] Fielding RA Vellas B Evans WJ Bhasin S Morley JE Newman AB . Sarcopenia: an undiagnosed condition in older adults. current consensus definition: prevalence, etiology, and consequences international working group on sarcopenia. J Am Med Directors Assoc. (2011) 12:249–56. doi: 10.1016/j.jamda.2011.01.003 21527165 PMC3377163

[7] Cruz-Jentoft AJ Baeyens JP Bauer JM Boirie Y Cederholm T Landi F . Sarcopenia: European consensus on definition and diagnosis: report of the European working group on sarcopenia in older people. Age Ageing. (2010) 39:412–23. doi: 10.1093/ageing/afq034 20392703 PMC2886201

[8] Michael A. Body composition at ct and risk of future disease. Radiology. (2023) 306:e222162. doi: 10.1148/radiol.222162 36165799 PMC9885345

[9] Marasco G Serenari M Renzulli M Alemanni LV Rossini B Pettinari I . Clinical impact of sarcopenia assessment in patients with hepatocellular carcinoma undergoing treatments. J Gastroenterol. (2020) 55:927–43. doi: 10.1007/s00535-020-01711-w 32748172 PMC7519899

[10] Schaffler-Schaden D Mittermair C Birsak T Weiss M Hell T Schaffler G . Skeletal muscle index is an independent predictor of early recurrence in non-obese colon cancer patients. Langenbeck's Archives of Surgery. (2020) 405:469–77. doi: 10.1007/s00423-020-01901-3 32504206 PMC7359173

[11] Laird BJ Kaasa S McMillan DC Fallon MT Hjermstad MJ Fayers P . Prognostic factors in patients with advanced cancer: a comparison of clinicopathological factors and the development of an inflammation-based prognostic system. Clin Cancer Res. (2013) 19:5456–64. doi: 10.1158/1078-0432.CCR-13-1066 23938289

[12] Medici F Rizzo S Buwenge M Arcelli A Ferioli M Macchia G . Everything you always wanted to know about sarcopenia but were afraid to ask: a quick guide for radiation oncologists (impact of sarcopenia in radiotherapy: the afraid project): 11. Current Oncol. (2022) 29:8513–28. doi: 10.3390/curroncol29110671 36354731 PMC9689889

[13] Huiskamp LFJ Chargi N Devriese LA May AM Huitema ADR de Bree R. The predictive value of low skeletal muscle mass assessed on cross-sectional imaging for anti-cancer drug toxicity: a systematic review and meta-analysis. J Clin Med. (2020) 9:3780. doi: 10.3390/jcm9113780 33238530 PMC7700117

[14] Baracos VE Reiman T Mourtzakis M Gioulbasanis I Antoun S. Body composition in patients with non–small cell lung cancer: a contemporary view of cancer cachexia with the use of computed tomography image analysis1234. Am J Clin Nutr. (2010) 91:1133S−7S. doi: 10.3945/ajcn.2010.28608C 20164322

[15] Nakamura R Inage Y Tobita R Yoneyama S Numata T Ota K . Sarcopenia in resected nsclc: effect on postoperative outcomes. J Thoracic Oncol. (2018) 13:895–903. doi: 10.1016/j.jtho.2018.04.035 29751134

[16] Martin L Birdsell L Macdonald N Reiman T Clandinin MT McCargar LJ . Cancer cachexia in the age of obesity: skeletal muscle depletion is a powerful prognostic factor, independent of body mass index. J Clin Oncol. (2013) 31:1539–47. doi: 10.1200/JCO.2012.45.2722 23530101

[17] Guo J Zheng C Xiao Q Gong S Zhao Q Wang L . Impact of anaemia on lung function and exercise capacity in patients with stable severe chronic obstructive pulmonary disease. BMJ Open. (2015) 5:e008295. doi: 10.1136/bmjopen-2015-008295 26450428 PMC4606425

[18] Buzby GP Mullen JL Matthews DC Hobbs CL Rosato EF. Prognostic nutritional index in gastrointestinal surgery. American J Surg. (1980) 139:160–7. doi: 10.1016/0002-9610(80)90246-9 7350839

[19] Fujiya K Kawamura T Omae K Makuuchi R Irino T Tokunaga M . Impact of malnutrition after gastrectomy for gastric cancer on long-term survival. Ann Surg Oncol. (2018) 25:974–83. doi: 10.1245/s10434-018-6342-8 29388124

[20] van Rijn-Dekker MI van den Bosch L van den Hoek JGM Bijl HP van Aken ESM van der Hoorn A . Impact of sarcopenia on survival and late toxicity in head and neck cancer patients treated with radiotherapy. Radiother Oncol. (2020) 147:103–10. doi: 10.1016/j.radonc.2020.03.014 32251949

[21] Mallet R Modzelewski R Lequesne J Mihailescu S Decazes P Auvray H . Prognostic value of sarcopenia in patients treated by radiochemotherapy for locally advanced oesophageal cancer. Radiation Oncol. (2020) 15:116. doi: 10.21203/rs.2.23302/v3 32443967 PMC7245030

[22] De Nardi P Giani A Maggi G Braga M. Relation between skeletal muscle volume and prognosis in rectal cancer patients undergoing neoadjuvant therapy. World J Gastrointest Oncol. (2022) 14:423–33. doi: 10.4251/wjgo.v14.i2.423 35317319 PMC8919003

[23] Dalal S Hui D Bidaut L Lem K Del Fabbro E Crane C . Relationships among body mass index, longitudinal body composition alterations, and survival in patients with locally advanced pancreatic cancer receiving chemoradiation: a pilot study. J Pain Symptom Manage. (2012) 44:181–91. doi: 10.1016/j.jpainsymman.2011.09.010 22695045 PMC3990439

[24] Kiyotoki T Nakamura K Haraga J Omichi C Ida N Saijo M . Sarcopenia is an important prognostic factor in patients with cervical cancer undergoing concurrent chemoradiotherapy. Int J Gynecol Cancer. (2018) 28:168–75. doi: 10.1097/igc.0000000000001127 29040185

[25] Katsui K Ogata T Sugiyama S Yoshio K Kuroda M Hiraki T . Sarcopenia is associated with poor prognosis after chemoradiotherapy in patients with stage III non-small-cell lung cancer: a retrospective analysis. Sci Rep. (2021) 11:11882. doi: 10.1038/s41598-021-91449-z 34088965 PMC8178326

[26] Olmez T Ofluoglu CB Sert OZ Keser SH Gulmez S Senger AS . The impact of sarcopenia on pathologic complete response following neoadjuvant chemoradiation in rectal cancer. Langenbeck's Archives of Surg. (2020) 405:1131–8. doi: 10.1007/s00423-020-01983-z 32902708

[27] Yamanouchi K Murakami S Sato A Ogawa S Shinagawa H Kamohara Y. Integrated evaluation of inflammatory, nutritional, and sarcopenia markers to predict survival in metastatic breast cancer patients. In Vivo. (2023) 37:811–7. doi: 10.21873/invivo.13146 36881066 PMC10026678

[28] Tuttle CSL Thang LAN Maier AB. Markers of inflammation and their association with muscle strength and mass: a systematic review and meta-analysis. Ageing Res Rev. (2020) 64:101185. doi: 10.1016/j.arr.2020.101185 32992047

[29] Gupta D Lis CG. Pretreatment serum albumin as a predictor of cancer survival: a systematic review of the epidemiological literature. Nutr J. (2010) 9:69. doi: 10.1186/1475-2891-9-69 21176210 PMC3019132

[30] Qian J Si Y Zhou K Tian Y Guo Q Zhao K . Sarcopenia is associated with prognosis in patients with esophageal squamous cell cancer after radiotherapy or chemoradiotherapy: 1. BMC Gastroenterol. (2022) 22:1–10. doi: 10.1186/s12876-022-02296-9 35501704 PMC9063365

[31] Baracos VE Arribas L. Sarcopenic obesity: hidden muscle wasting and its impact for survival and complications of cancer therapy. Annal Oncol. (2018) 29:II1–9. doi: 10.1093/annonc/mdx810 29506228

[32] Li Y Wang WB Yang L Wang QY Dai J Xia L . The combination of body composition conditions and systemic inflammatory markers has prognostic value for patients with gastric cancer treated with adjuvant chemoradiotherapy. Nutrition. (2022) 93:111464. doi: 10.1016/j.nut.2021.111464 34678715

[33] Stangl-Kremser J D'Andrea D Vartolomei M Abufaraj M Goldner G Baltzer P . Prognostic value of nutritional indices and body composition parameters including sarcopenia in patients treated with radiotherapy for urothelial carcinoma of the bladder. Urol Oncol: Seminar Orig Investig. (2019) 37:372–9. doi: 10.1016/j.urolonc.2018.11.001 30578161

[34] Xu YJ Li P Hu X Wang J Ma HL Chen M. [Dosimetric comparison of the helical tomotherapy, intensity-modulated radiotherapy and volumetric-modulated arc therapy in radical radiotherapy for esophageal cancer]. Zhonghua Yi Xue Za Zhi. (2019) 99:3260–5. doi: 10.3760/cma.j.issn.0376-2491.2019.41.012 31694123

